# Relapse after severe acute malnutrition: A systematic literature review and secondary data analysis

**DOI:** 10.1111/mcn.12702

**Published:** 2018-10-18

**Authors:** Heather C. Stobaugh, Amy Mayberry, Marie McGrath, Paluku Bahwere, Noël Marie Zagre, Mark J. Manary, Robert Black, Natasha Lelijveld

**Affiliations:** ^1^ Food, Nutrition, and Obesity Policy and Research Team RTI International Research Triangle Park North Carolina; ^2^ No Wasted Lives Team Action Against Hunger London UK; ^3^ Emergency Nutrition Network Oxford UK; ^4^ Valid International Oxford UK; ^5^ Centre de Recherche en Epidémiologie, Biostatistique et Recherche Clinique, Ecole de santé publique Université Libre de Bruxelles City of Brussels Belgium; ^6^ West and Central Africa Regional Office UNICEF West and Central Africa Regional Office Dakar Senegal; ^7^ Department of Pediatrics Washington University in St. Louis St. Louis Missouri; ^8^ Institute for International Programs Johns Hopkins Bloomberg School of Public Health Baltimore Maryland; ^9^ Centre for Global Child Health, The Hospital for Sick Children Toronto Canada

**Keywords:** community‐based management of acute malnutrition, outpatient therapeutic programme, post‐discharge outcomes, relapse, severe acute malnutrition, wasting

## Abstract

The objectives of most treatment programs for severe acute malnutrition (SAM) in children focus on initial recovery only, leaving post‐discharge outcomes, such as relapse, poorly understood and undefined. This study aimed to systematically review current literature and conduct secondary data analyses of studies that captured relapse rates, up to 18‐month post‐discharge, in children following recovery from SAM treatment. The literature search (including PubMed and Google Scholar) built upon two recent reviews to identify a variety of up‐to‐date published studies and grey literature. This search yielded 26 articles and programme reports that provided information on relapse. The proportion of children who relapsed after SAM treatment varied greatly from 0% to 37% across varying lengths of time following discharge. The lack of a standard definition of relapse limited comparability even among the few studies that have quantified post‐discharge relapse. Inconsistent treatment protocols and poor adherence to protocols likely add to the wide range of relapse reported. Secondary analysis of a database from Malawi found no significant association between potential individual risk factors at admission and discharge, except being an orphan, which resulted in five times greater odds of relapse at 6 months post‐discharge (95% CI [1.7, 12.4], *P* = 0.003). The development of a standard definition of relapse is needed for programme implementers and researchers. This will allow for assessment of programme quality regarding sustained recovery and better understanding of the contribution of relapse to local and global burden of SAM.

**Key messages**

- Relapse following treatment of severe acute malnutrition (SAM) is poorly defined and scarcely measured across programs and research.
- Reported relapse ranges from 0% to 37% of children following SAM treatment, with the highest proportions occurring within 6 months post‐discharge. The data across studies are largely not comparable due to different treatment protocols, various follow‐up periods, and inconsistent reporting of relapse as a point prevalence (not cumulative), cumulative prevalence, and incidence rate.
- Lower anthropometric measurements on admission to and discharge from SAM treatment are consistent risk factors for relapse. Illness is frequently observed at the time of relapse.
- A standardized definition of relapse and a maximum acceptable relapse rate are needed.

## 1 INTRODUCTION

Around 17 million children worldwide suffer from severe acute malnutrition (SAM), defined as having a weight‐for‐height *z*‐score (WHZ) less than −3 *SD* or a mid‐upper arm circumference (MUAC) less than 115 mm (United Nations Children's Fund, World Health Organization, & World Bank Group, 2017). Estimates reveal that worldwide numbers of children who suffer from acute malnutrition have decreased very little (only 11% over the past 20 years), particularly when compared with progress made in reducing other malnutrition indicators, such as stunting (Annan, Webb, & Brown, 2014). The immediate consequences of SAM are life threatening, as a child with SAM is approximately nine times more likely to die than a non‐malnourished child (Black et al., 2008).

Although most of the research conducted around SAM addresses the causes, short‐term consequences, and treatment methods for achieving immediate recovery, little is known about the overall health and nutrition of children following discharge. A small body of evidence is emerging from the few studies that followed children after treatment for SAM, demonstrating poor post‐discharge outcomes after initial recovery including mortality, morbidity, and functional implications (Bahwere, Mtimuni, Sadler, Banda, & Collins, 2012; Lelijveld et al., 2016). One of the most immediate outcomes that needs to be understood and addressed is relapse to SAM. To find appropriate, scalable solutions that can tackle relapse, there is need to identify the overall burden of relapse in different contexts as well as better understand potential risk factors and consequences associated with relapse. Although standard community‐based management of acute malnutrition (CMAM) programme report forms based on the Sphere Minimum Standard guidelines do include space to record relapse up to 2 months post‐discharge, without standardized guidance on how to capture relapse accurately or an evidence‐base to prove the importance of allocating resources to do so, it is infrequently reported.

In this study, we aimed to systematically review current literature and conduct secondary data analyses on a dataset from Malawi to better understand relapse rates in children following recovery from SAM treatment.

## 2 METHODS

### 2.1 Search strategy

This literature review focused on retrieving and analysing peer reviewed publications as well as other programme reports, evaluations, and grey literature. This was conducted through three main search components. The first consisted of reviewing two previously conducted literature reviews (Akparibo, Lee, & Booth, 2017; Bahwere, 2016) to include only articles reporting relapse within 18 months of discharge from SAM treatment. The second search component of this review included a computerized search of PubMed and Google Scholar between January 1, 2015, and October 15, 2017, designed to update the previous reviews. The following search terms were used: “protein energy malnutrition” (Mesh), “severe acute malnutrition” (Mesh), “acute malnutrition,” “malnourish*,” “undernourish*,” “severe wasting,” “severely wasted,” “severely undernourished,” “severe undernutrition,” “child*,” “infant*,” “preschool*,” “toddler*,” “relapse,” “readmission,” “after recovery,” “after discharge,” “after cured,” “post‐discharge,” “post‐SAM,” or “long term.” Reference lists of articles were also screened for further relevant publications. To identify ongoing research and other relevant grey literature, the websites of NGOs, donors, and knowledge‐sharing platforms such as the Emergency Nutrition Network were searched. Co‐authors of this paper inquired to their respective networks of researchers in order to identify further unpublished reports or ongoing studies, as well as any relevant datasets for secondary data analysis. All searches were limited to studies published in English, Spanish, or French.

### 2.2 Study selection

Relevant articles were obtained and included in this review if they met inclusion and exclusion criteria based on three main items—population, intervention, and outcome. This review included studies if they involved children under the age of 5 years old who received treatment for SAM. Only studies that used definitions of SAM by anthropometric measurements based upon the National Center for Health Statistics child growth references, World Health Organization (WHO) child growth standards, and/or MUAC were included.

The included literature consisted of interventions specifically designed to treat children with SAM, namely, inpatient care using therapeutic milks and outpatient care using ready‐to‐use therapeutic foods. There were no parameters around location or whether the context was considered a humanitarian emergency or development setting. The main outcome of interest in this review was relapse to SAM following the treatment of SAM. The concept of relapse is not well defined in the literature; the distinction between “relapse” and a new episode of SAM is especially difficult to differentiate. We therefore set a generous time limit of 18 months post‐discharge as possible relapse for the purposes of the search.

### 2.3 Screening process and data extraction

We screened all publications identified in the two previous literature reviews and updated electronic searches by reading the titles and abstracts to determine initial relevance. After removing duplications, a second screening process consisted of reading full texts of the remaining articles and those that met the inclusion/exclusion criteria were retained. Data were extracted into an excel file that included information on authors, date of publication, county, title, type of paper, study design, type of intervention or programme to treat SAM, admission and discharge criteria for the treatment of SAM, follow‐up length and schedule, relapse rates, and other findings and comments.

We felt that applying a systematic quality assurance checklist would be too limiting given such few studies regarding post‐SAM relapse exist. Therefore, we aimed to include all literature that may provide relevant information on the topic. We evaluated the strengths and weaknesses of each research article and programme report independently to ensure that quality of study design was considered when interpreting results.

### 2.4 Secondary data analysis

For the secondary data analysis, one dataset was made available: a retrospective follow‐up of children treated in a CMAM programme in Dowa, Malawi, between 2002 and 2005, with follow‐up periods ranging from 1 to 32 months post‐discharge (Bahwere et al., 2012). We assessed the association of child and household characteristics with odds of relapse to SAM up to 6 and 12 months post‐discharge from treatment, using logistic regression and STATA 14 software. A long list of potential exposure variables was created prior to sourcing secondary data, including child anthropometry during treatment, child health history, family characteristics, and household characteristics. All available variables from this list of potential exposures were included in analysis.

## 3 RESULTS

### 3.1 Search results

A flowchart detailing the process of study selection is shown in Figure S1 **.** Ultimately, 26 articles and reports were included in the final qualitative synthesis, including 21 original research studies, four programme evaluation reports, and one coverage survey (Table 1). Of the published studies, 13 were prospective longitudinal cohorts; three were cross‐sectional studies; three retrospective cohorts; and two were secondary data analysis.

**Table 1:** Characteristics of articles and reports identified in the review as specifically addressing relapse following severe acute malnutrition treatment

First author/date/country	Type of paper/study design	Type of programme	Admission criteria (SAM definition)	Discharge criteria	Follow‐up length	Follow‐up schedule	Indicator reported	Relapse rate	Findings, comments, and limitations			
Ashraf/2012/Bangladesh	Original research/prospective cohort	Day care	WHZ < −3 WAZ < −3 oedema	WHM ≥ 80%	6 months	Weekly (2 visits), fortnightly (5 visits), quarterly (3 visits)	Incidence	17.8%	Observed persistent stunting, high prevalence of illness in first 3 months, study experienced high drop‐out rate			
Bahwere/2008/Malawi	Original research/cross‐sectional	CMAM	WHM < 70% MUAC < 110 mm oedema	WHM ≥ 80%	15.6 months (median length)	3, 12 months	Point prevalence	3% (35.7% for HIV+ and 2% for HIV−)	Recommend more RUTF for HIV+ children, continued feeding for HIV+ children, and link CMAM model with HIV treatment			
Beau/1993/Senegal	Original research/cross‐sectional	Facility‐based	WHZ < −2 oedema	WHM ≥ 80%	12 months	Once at endpoint	Point prevalence	10.1%	Suggests return to an unfavourable environment and poor adoption of nutrition counselling messages explain high relapse rates			
Begashaw/2013/Nigeria	Coverage survey	CMAM	WHZ < −3 MUAC < 115 mm oedema	MUAC ≥ 125 mm	Unclear; mothers were asked if child had previously been admitted and discharged	N/A (readmissions)	Unclear	25%	Observed high prevalence of illness at time of relapse; rates rely of caregivers' report of prior treatment			
Bhandai/2017/India	Original research/prospective cohort	CMAM	WHZ < −3 oedema	WHZ ≥ −2	16 weeks	Once at endpoint	Point prevalence	37.4% to SAM, 48% to MAM (42.4% local diet, 40.7% conventional RUTF, 29.2% local RUTF)	RUTF resulted in lower relapse rates than fortified home‐based diet		
Binns/2016/Malawi	Original research/prospective cohort	CMAM	MUAC < 115 mm oedema	MUAC ≥ 125 mm for 2 consecutive weeks	3 months	Fortnightly	Incidence	1.9%	MUAC deemed an appropriate discharge criterion; early detection of SAM may reduce relapse. Cure rate for initial treatment programme was low (63%) due to early discharge. Children in SFP during follow‐up period			
Burrell/2017/Gambia	Original research/retrospective secondary data analysis	CMAM	WHZ < −3 MUAC < 115 mm	MUAC ≥ 125 mm WHZ ≥ −2	1–4 weeks	N/A (readmissions)	Unclear	6% (7.1% and 3.8% for MUAC and WHZ discharge)	No statistical difference in relapse rates between discharge criteria using MUAC versus WHZ; no established follow‐up procedure; all relapses were defined as self‐referring readmissions. Likely an underestimation of true relapse			
Burza/2016/India	Original research/prospective cohort	CMAM	MUAC < 115 mm oedema	MUAC ≥ 120 mm for 2 consecutive weeks	18 months	Quarterly	Point prevalence	9.1%, 2.9%, 2.1%, 2.8%, and 0% at 3, 6, 9, 12, and 18 months, respectively	Associations with relapse include: seasonality, use of health services, lower standard of living, less time outside the programme, low HAZ at time of discharge			
Ciliberto/2005/Malawi	Original research/prospective cohort	CMAM versus facility‐based	WHZ < −3 oedema	WHZ > −2	6 months	Once at endpoint	Point prevalence	6.9% (6.2% and 10.6% for home‐RUTF and inpatient)	Receiving home‐based RUTF is associated with lower risk of relapse		
Cuneo/2017/Haiti	Original research/retrospective analysis	CMAM	WHZ < −3 MUAC < 115 mm	Operationally: WHZ > −1 for 2 consecutive weeks; data analysis of “cured”: 15% weight gain	Unclear	N/A (readmissions)	Unclear	6% over the total 5 years (5%, 3%, 0%, 8%, 4%, 20% for each year of 2009–2013, respectively); only 56% cured at discharge	Low cure rate at discharge reflects programme quality, which likely impacts relapse rates		
Dani/2016/India	Original research/prospective cohort	CMAM‐providing locally made therapeutic food for 90 days	WHZ ≤ −3 WAZ ≤ −3	Unclear	6 months	Once at endpoint	Point prevalence	3% SAM and 11% severely underweight	Higher relapse rates to underweight may be due to stunting following SAM		
Grellety/2017/DRC	Original research/prospective cohort	CMAM + cash transfer program	WHZ < −3 MUAC < 115 mm oedema	MUAC ≥ 125 mm or WHZ ≥ −1.5 for 2 consecutive weeks	3–5 months (6 months after admission)	Monthly	Incidence	12.6% SAM, 28% MAM, (CMAM + CTP: 3% SAM and 13% MAM; CMAM only: 11% SAM and 44% MAM)	An unconditional cash transfer in combination with CMAM led to significantly lower relapse, time of follow‐up after discharge varied between participants		
Khanum/1998/Bangladesh	Original research/prospective cohort	Facility based, day‐care, domiciliary	WHM < 60% oedema	WHM ≥ 80%	12 months	Fortnightly	Incidence	1%	All study participants lived <10 km of the health facility; access to healthcare services may explain low relapse rates		
Magnin/2017/Madagascar	Original research/retrospective analysis	CMAM	WHZ < −3	WHZ > −1	1 year	Once at endpoint	Point prevalence	1%	Being younger, higher admission WHZ, and use of chlorine water treatment increased likelihood of maintaining recovery; when children were lost to follow‐up, data on other children who had participated in the programme were used		
Mengesha/2016/Ethiopia	Original research/retrospective analysis	CMAM	MUAC < 110 mm	MUAC ≥ 110 and 15% weight gain	2 months	N/A (readmissions)	Point prevalence	22%	Low admission MUAC criteria and % weight gain as discharge criteria may explain higher relapse rates		
Nyirenda/2010/Ethiopia	Programme evaluation	CMAM	WHM < 70% MUAC < 110 mm oedema	WHM ≥ 85% or 15% weight gain for 2 consecutive weeks	Unclear	N/A (readmissions)	Unclear	1%, 1.3%, 1.8% in 2007, 2008, 2009, respectively	No established follow‐up procedure; all relapses were defined as self‐referring readmissions. Likely an underestimation of true relapse.	
Pecoul/1992/Niger	Original research/prospective cohort	Facility based	WHZ < −3 oedema	WHZ ≥ −3	3–18 months	Unclear	Point prevalence	0%	Small sample size, outdated treatment protocol, high mortality and default rate during treatment, no follow‐up occurred between discharge and 3 months	
Perra/1995/Guinea Bissau	Original research/prospective cohort	Facility‐based	WAM < 60%	Unclear	18 months	Unclear	Incidence	1%	Children treated for SAM had better outcomes than those who had not been treated; response to prior study saying treatment was a waste. One cross‐sectional survey at 18 months follow‐up is likely to miss relapses that take place closer to the time of discharge.	
Querubin/2006/Sudan	Programme evaluation	Facility based and CMAM	WHM < 70% MUAC < 110 mm	WHM ≥ 80% or MUAC ≥ 120 for 2 consecutive weeks	Unclear	Unclear, readmission?	Unclear	0%	Report was written using programme monitoring data and does not include much detail regarding follow‐up procedures or duration			
van Roosmalen‐Weibenga/1987/Tanzania	Original research/prospective cohort	Facility‐based	Unclear	Unclear	12 months	Unclear	Point prevalence	13%	Difficult to compare results as treatment protocols are outdated			
Singth/2016/India	Original research/prospective longitudinal cohort	Facility based	WHZ < −3 MUAC < 115 mm oedema	15% weight gain	Approximately 1.5 months	1 week, then every 15 days	Point prevalence	4.9% total (8%, 3%, 5% for 0–6, 7–24, 25–59 months, respectively)	Suggested better integration between facility‐based and community‐based treatment to reduce relapse			
Somasse/2015/Burkina Faso	Original research/prospective longitudinal cohort	CMAM	MUAC < 110 mm oedema	WHZ > −2	12 months	Quarterly	Point prevalence	11% SAM; 16% AM	High lost to follow‐up (34%), which may lead to underestimation of relapse; MUAC upon discharge below 125 mm, no oil/fat consumption, and incomplete vaccination were all associated with relapse			
Tadese/2017/Ethiopia	Original research/prospective longitudinal cohort	CMAM	MUAC < 110 mm oedema	15% weight gain	14 weeks after admission	4, 8, and 14 weeks after admission (about 2–6 weeks after discharge)	Point prevalence	Recovered: 30% to SAM and 50% to MAMtotal: 35% to SAM and 38% to MAM	Many children discharged prior to anthropometric recovery; proportion of children readmitted was significantly higher among children with most severe degree of wasting on admission			
Taylor/2002/Sudan	Programme evaluation/programme M&E	CMAM	WHZ < −3 oedema	WHM > 75% for 4 consecutive weeks	Unclear	Unclear, readmission?	Unclear	1%	Report was written using programme monitoring data and does not include much detail regarding follow‐up procedures or duration			
Tsinuel/2015/Ethiopia	Original research/prospective cohort	CMAM	MUAC < 110 mm oedema	MUAC > 110 mm and 20% weight gain for 2 consecutive weeks	12 months	Monthly	Incidence	By then end, 15% to SAM in post‐SAM, 1% in healthy controls	Post‐SAM children had higher risk for AM than controls; MUAC, HAZ, food security, and IYCF were associated with relapse; discharge using % weight gain may increase relapse rates	
UNICEF/2012/Kenya	Programme evaluation/programme M&E	Facility‐based and CMAM	WHZ < −3 MUAC <115 mm	Unclear	12 months?	Unclear	Unclear	6.1% inpatient, 3.2% outpatient	Health workers stated that sharing RUTF negatively affects relapse.			

*Note*. AM: acute malnutrition; CMAM: community‐based management of acute malnutrition; CTP: cash transfer programme; HIV: human immunodeficiency virus; MAM: moderate acute malnutrition; M&E: monitoring and evaluation; MUAC: mid‐upper arm circumference; RUTF: ready‐to‐use therapeutic food; SAM: severe acute malnutrition; SFP: supplementary feeding programme; WAZ: weight‐for‐age *z*‐score; WHM: weight‐for‐height percent of median; WHZ: weight‐for‐height *z*‐score.

### 3.2 Definition of relapse and relapse rates

The definition of relapse varied across studies and programme reports. There was also differing anthropometric admission and discharge criteria, duration of follow‐up, frequency of data collection points, and data collection methods (i.e., active follow‐up visits vs. readmission based on passive, self‐referrals; Table 1). Generally, relapse was defined as a child presenting at least once with SAM within a specified time period following discharge (or default) from SAM treatment. Anthropometric criteria for relapse typically coincided with the anthropometric admission criteria for initial SAM treatment but sometimes also included the development of moderate acute malnutrition (MAM). No standard length of time was used, ranging from 1 week to 18 months after discharge across the studies. Most of the original research studies conducted active follow‐up visits, whereas evaluations and programme reports tended to report relapse based on readmission.

**Figure 1:**
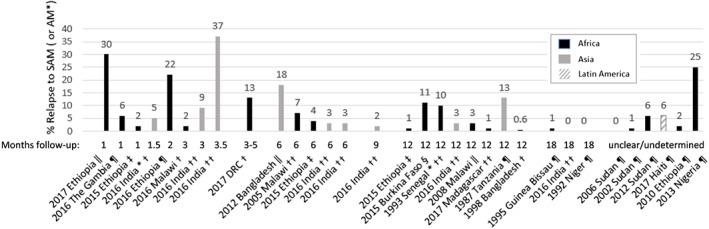
Relapse rates presented in literature according to the duration of follow‐up after discharge from treatment of SAM. AM: acute malnutrition; SAM: severe acute malnutrition. When relapse rates were disaggregated between those who met discharge criteria and those who did not, rates of those who met discharge criteria were included. However, it was not always clear if all children included in the relapse rates were discharged as recovered. Also, some studies included relapse to AM where others included only relapse to SAM. Lastly, rates also vary between those that are point prevalence or cumulative; thus, relapse rates in Figure 1 are not fully comparable. Data measured: † fortnightly, ‡ monthly, § quarterly, ‖ varied, ¶ readmission/unclear, and †† once at endpoint. * Studies with relapse defined as relapse to AM (including MAM or SAM). Such studies that report relapse back to MAM or SAM are those where children are discharged at a point in which they are deemed “not malnourished” and therefore a “relapse” to MAM is indeed a true regression in nutritional status

The proportion of children who relapsed following treatment for SAM spanned from 0% to 37% at various points following discharge (Figure 1). (The denominators to these proportions varied, including children discharged and/or defaulted from SAM treatment programs.) Relapse tended to occur more frequently during the first 6 months following discharge. For example, a 2016 study in India that followed children on a quarterly basis for 18 months after discharge found that children were more likely to relapse in the first 3 months (9.1%) versus 6 months (2.9%), 9 months (2.1%), 12 months (2.8%), and 18 months (0%; Burza et al., 2016). A 2015 longitudinal study in Ethiopia demonstrated the probability of experiencing a new episode of acute malnutrition (AM) was 26% and 7.5% for 6 and 12 months, respectively (Tsinuel, Alemseged, Philips, Paluku, & team, 2015). When including both MAM and SAM in the definition of relapse, the proportion of relapse increases dramatically, from 38% to 86% at 3 months in India (Bhandari et al., 2016), 30% to 80% over 3.5 months in Ethiopia (Tadesse, Worku, Berhane, & Ekstrom, 2017), 13% to 41% over 6 months in the Democratic Republic of Congo (Grellety et al., 2017), and 15% to 44% over 12 months in Ethiopia (Tsinuel et al., 2015) for relapse to SAM and AM, respectively.

In this review, only one study included a true control group of non‐malnourished counterparts for which to compare excess relapse (Tsinuel et al., 2015). This 2015 longitudinal study in Ethiopia followed children after SAM treatment as well as matched nonwasted community controls for 1 year and found 15% relapse in post‐SAM group yet 1.2% of the control children became severely malnourished in the same time period. The incidence rate was 1.27 and 0.09 per 100 person‐months for post‐SAM and controls, respectively (Tsinuel et al., 2015).

Indicators of relapse were reported as a mixture of point prevalence (i.e., how many children were in a state of relapse at one specific point in time) and a cumulative proportion or incidence rate (i.e., how many children relapsed or how many relapses occurred throughout a certain period of time). Those who reported relapse as a cumulative proportion or incidence rate tended to be higher than point prevalence, as they likely captured more relapse simply by the nature of more frequent data collection points. For example, when post‐discharge children are only measured at one point in time, such as at 12 months after discharge, it is likely that many children relapsed and recovered prior to that 12‐month follow‐up time that are not accounted for in this statistic. Also, point prevalence does not account for multiple episodes of relapse that occur per child prior to that point in time. The difference between reporting relapse as point prevalence and cumulative incidence is seen in the 2015 Ethiopian study where both indicators were calculated. In this study, reported relapse is 1% as a point prevalence at 12 months post‐discharge (meaning only 1% of children was in a state of relapse at the time of 12 months post‐discharge) and yet relapse is 15% cumulative incidence over the course of the entire 12 months (Tsinuel et al., 2015).

Consistently, studies and programme evaluations reported that children who defaulted or were discharged prior to reaching recommended anthropometric discharge criteria had higher risk for relapse (Akparibo et al., 2017; Ashworth, 2001; Binns et al., 2016; Burza et al., 2016; Pecoul, Soutif, Hounkpevi, & Ducos, 1992; Tadesse et al., 2017). In Niger, those who defaulted during treatment had 7.1 times higher risk of death and were more likely to relapse at 3 months than those who were discharged as recovered (Pecoul et al., 1992). A 2016 study in India observed relapse as high as 52% of children who defaulted from SAM treatment (Burza et al., 2016). Including defaulters in the definition of relapse likely inflates the proportion of relapse due to the inability to determine whether those defaulters ever reached a state of recovery (and thus truly “relapsed” back to being malnourished again) or remained malnourished throughout the time after leaving the treatment programme to the follow‐up point. The latter case is not a true relapse, rather a prolonged case of unresolved acute malnutrition.

The strongest, most consistent risk factor associated with relapse was having lower anthropometric measurements upon admission to and discharge from treatment of SAM (Ashraf et al., 2012; Bahwere et al., 2008; Beau, 1993; Begashaw, 2013; Binns et al., 2016; Khanum, Ashworth, & Huttly, 1998; Somasse, Dramaix, Bahwere, & Donnen, 2015; Tadesse et al., 2017; Tsinuel et al., 2015). Illness was observed at the time of relapse in eight studies (Ashraf et al., 2012; P. Bahwere et al., 2008; Begashaw, 2013; Binns et al., 2016; Khanum et al., 1998; Somasse et al., 2015; Tadesse et al., 2017; Tsinuel et al., 2015). Several authors suggested that children who are discharged as recovered from SAM treatment based on anthropometrics alone may not have experienced full immunologic recovery, leaving them susceptible to infection and subsequent relapse. Although rarely measured, micronutrient deficiencies were not associated with relapse (Tsinuel et al., 2015). Poor linear growth and stunting were also consistently observed post‐discharge (Paluku Bahwere, 2016; Lelijveld et al., 2016; Tsinuel et al., 2015). Data are mixed regarding significant associations between relapse and household‐level factors, such as socio‐economic status, feeding practices, and sanitary living conditions (Burza et al., 2016; Kerac et al., 2014; Magnin, Stoll, Voahangy, & Jeannot, 2017; Somasse et al., 2015; Tsinuel et al., 2015). Results are inconclusive regarding the effect of seasonality and food security on relapse (Burza et al., 2016; Tsinuel et al., 2015). Unconditional cash transfers during and following the treatment of SAM led to a decrease in relapse rates (Grellety et al., 2017).

### 3.3 Secondary data analyses

The database of children from a CMAM programme in Dowa, Malawi contained 1,361 records with a mean follow‐up period of 15 months (range 1–32 months). Stratifying the data by length of follow‐up found that 14% (16/118) and 12% (33/269) of children relapsed by 6 and 12 months post‐discharge, respectively. Proportion of relapse is similar when presenting only those discharged as recovered: 12% (14/113) and 13% (29/225), respectively. Loss to follow‐up and/or survivor bias, may be underestimating the relapse rate, especially among those who defaulted from treatment. There was no significant difference in odds ratio of relapse at 6 nor 12 months when regressed against age at admission, sex, oedema at admission, diarrhoea at admission, fever at admission, cough at admission, MUAC at admission, mother not alive, father not alive, attendance to supplementary feeding program, or MUAC at discharge, respectively. Being an orphan was associated with five times greater odds of relapse at 12 months' post‐discharge (95% CI [1.7, 12.4], *P* = 0.003; Table S1).

## 4 DISCUSSION

Results of this review have highlighted that relapse is poorly defined and scarcely measured across programs and research alike. The lack of a standard definition of relapse following the treatment of SAM has led to relapse rates that are largely not comparable, with varying follow‐up time periods and inconsistent reporting between point prevalence, cumulative prevalence, and incidence indicators.

Different treatment protocols, such as varying admission and discharge criteria, likely add to the wide range of relapse rates. For example, discharge based on percent weight gain has been shown to be less effective than MUAC or WHZ cut‐offs in reaching a true recovery (Dale, Myatt, Prudhon, & Briend, 2013), and using a lower MUAC cut‐off for admission criteria (i.e., MUAC <110 mm) leads to the selection of more severely malnourished children, who may be at higher risk for relapse (Stobaugh et al., 2017). Adhering to the WHO's recommended admission and discharge criteria and attempting to reduce default rate may help to lower relapse. Innovative approaches such as teaching mothers to screen children's MUAC, the use of community health workers to treat acute malnutrition, and expanded admission criteria which include MAM children in therapeutic treatment, may facilitate lower risk of relapse by improving default rates and early identification of new cases (Ale et al., 2016; Maust et al., 2015). This review also highlights the importance of early identification of SAM cases such that children receive treatment before experiencing a more severe degree of physiological insult and risk of relapse increases. However, more research is needed to determine the effects of these innovations on relapse rates.

Continuity of care following initial treatment for SAM is crucial for children to reach and maintain lasting recovery. Continued nutritional support, potentially in the form of a supplementary feeding programme, may contribute to lower relapse rates, especially if national protocols call for children to be discharged at MUAC equals 115 mm or WHZ equals −3 (Mengesha, Deyessa, Tegegne, & Dessie, 2016). Continued nutritional support may help to progress anthropometric status to the WHO recommended discharge cut‐off of MUAC equals 125 mm or WHZ equals −2. However, it is acknowledged the SFPs vary in design and quality, and although uptake of SFP services reduced odds of relapse in our secondary data analysis, it was not statistically significant. Still, having a higher MUAC and WHZ upon discharge has seen to be protective against relapse in other analyses.

Addressing co‐morbidities by incorporating programmatic linkages to healthcare services and the promotion of appropriate health seeking behaviours may also help to improve the sustainability of recovery following SAM. Post‐discharge mortality is shown to be highest among HIV+ children, demonstrating the need to better integrate antiretroviral treatment and possibly identify therapeutic foods that might cater to the specific nutritional needs of malnourished children with HIV (Kerac et al., 2014). In this review, one of the studies with the lowest proportion of relapse, at 0.6%, occurred where study participants lived near a health facility, with 90% of the children visiting the facility at least once, and 53% receiving antibiotics at least once over the course of the 12‐month follow‐up period (Khanum et al., 1998). This suggests that routine follow‐up with access to healthcare services among this high‐risk population of post‐SAM children can lead to timely referrals and treatment for illness that may otherwise leave children highly susceptible to morbidity and relapse. Greater availability and quality of follow‐up home visits, integration of primary health care services, and provision of other welfare support post‐discharge, such as cash transfers, may therefore improve relapse rates.

The development of a standard definition of relapse is needed for researchers and programme implementers to better understand the burden of relapse and assess the sustainability of recovery when using current treatment protocols. For research purposes, such standardized reporting of relapse should include the following: a definition of timescale, data collection methods (i.e., systematic tracking vs. passive readmissions), frequency of data collection points within the recommended follow‐up period, clarification for reporting the severity of relapse episodes (i.e., disaggregating between relapse to SAM vs. AM), and disaggregation of those who met the discharge criteria from those who defaulted. Programme reports and studies that do not disaggregate relapses of defaulters from those who reached discharge criteria may need to be interpreted with caution.

Given that this review found the highest risk for relapse occurs during the first 6 months following discharge, and studies measuring relapse following recovery from MAM showed similar timing of relapse post‐discharge (Chang et al., 2013; Stobaugh et al., 2017), fortnightly follow‐up for the first 3 months is ideal for identifying relapse. Further, monthly follow‐up between 3 and 6 months is also recommended with analysis disaggregated between the first 3 months and the following 3–6 months. Follow‐up of discharged children should be systematically and actively pursued, in addition to referrals from other service providers and readmissions based on self‐referrals at any time during the follow‐up period.

It is also important to distinguish between absolute versus excess relapse (Bahwere, 2016). The absolute relapse rate should be compared with the regular incidence of SAM in nonpreviously malnourished children over the same time period in order to estimate the excess burden of relapse. Where possible, longitudinal studies should include a comparison group of nonpreviously wasted children from the same community who are followed alongside post‐SAM children to serve as a control group. This leads to the calculation of expected events for comparison with observed events. In the least, both researchers and programme implementers should present relapse or readmission rates in the context of SAM prevalence in order to better understand the excess acute malnutrition resulted by relapse. The 2015 Ethiopia study's follow‐up procedures and use of a nonpreviously wasted control groups serve as a strong example for how to capture excess relapse well (Tsinuel et al., 2015).

Such rigorous follow‐up procedures may not be feasible in routine or emergency CMAM programs where resources are limited. In such circumstances, caregivers of discharged children should be requested to return to the CMAM facility at 1, 3, and 6 months post‐discharge to monitor the child's progress and reassess for relapse. Although this would likely lead to additional screenings at CMAM facilities, children who relapse will be caught earlier in their regression and thus, in theory, require fewer resources to retreat than otherwise waiting until the child's nutritional status deteriorates significantly. If possible, routine CMAM programs should utilize community health workers or volunteers to visit the homes of recovered children at 1, 3, and 6 months post‐discharge if caregivers fail to voluntarily bring the child back to the facility for post‐discharge reassessment. This would allow for more accurate relapse rates than solely relying on voluntary returns. At a minimum, routine programs should emphasize active‐case finding at the community level for both nonpreviously and previously malnourished children, which in turn would not only improve timely identification of new cases but also the capture of relapse, where data records allow.

There is no standard in the literature to assess what rate of relapse is high, low, or acceptable. Evidence is needed to better understand the consequences of relapse in order to determine a target rate of relapse under which treatment programs should aspire to remain. Once there is further data, a maximum acceptable relapse or readmission rate could be added to the Sphere Minimum Standards (*The Sphere Project Handbook*, 2011) and included as a regularly collected indicator of programme quality in the treatment of SAM. Also, post‐discharge mortality should be taken into account in order to determine how survivor bias may effect relapse rates, especially in contexts where HIV is prevalent, and the mortality rate is particularly high among post‐discharge SAM children. High mortality may lend to a lower relapse rate and give the false impression that post‐discharge outcomes are not poor. Additional standards for programme monitoring and quality indicators should include minimum community screenings and follow‐up visits of discharged children.

Research, programming, and policy need to shift the focus of acute malnutrition from not only achieving immediate recovery to achieving sustained recovery and improving other long‐term outcomes of children with SAM. Further research is necessary to define relapse and better understand the risk factors and consequences associated with relapse. Studies are warranted regarding the following: identification of children at high risk of relapse, different discharge criteria and its effect on relapse, innovative methods for improving early identification of acute malnutrition, interventions and innovative formulas for therapeutic foods that improve immune function immediately after discharge, and other interventions to reduce relapse in high‐risk children.

## CONFLICTS OF INTEREST

The authors declare that they have no conflicts of interest.

## CONTRIBUTIONS

MM, PB, NMZ, MJM, and RB conceived the study and determined the overall focus of the review. HCS, NL, and AM designed the parameters of the review. HCS conducted the literature search. NL conducted the secondary data analyses. HCS and NL jointly wrote the first draft of the manuscript. All authors edited the manuscript and approved its final contents. HCS has primary responsibility for the paper's final content.

## Supporting information

Table S1. Results of logistic regression assessing factors associated with relapse up to 6 or 12 months after discharge across two datasets

Figure S1. Flow chart of literature review methods
