# Supplementary material for: Relapse after severe acute malnutrition: A systematic literature review and secondary data analysis
Source: Matern Child Nutr. 2018 Oct 18;15(2):e12702. doi: 10.1111/mcn.12702 (PMC6587999; doi:10.1111/mcn.12702)
Supplement: Supplementary file 1 — Table S1. Results of logistic regression assessing factors associated with relapse up to 6 or 12 months after discharge across two datasets [file MCN-15-e12702-s002.docx]

**Supplemental Table 1.** Results of logistic regression assessing factors associated with relapse up to 6 or 12 months
after discharge across 2 datasets^a^

|  | **CMAM Dowa database* followed-up to 6 months post-discharge (n=118)** | | |  | **CMAM Dowa database*followed-up to 12 months  post-discharge (n=269)** | | |  |
| --- | --- | --- | --- | --- | --- | --- | --- | --- |
| **Child characteristic** | **OR** | **95% CI** | ***P*** |  | **OR** | **95% CI** | ***P*** |  |
| Child age (months) | 1.01 | 0.98 to 1.0 | 0.53 |  | 1.01 | 0.9 to 1.0 | 0.61 |  |
| Older than 2 years at admission | 2.18 | 0.6 to 7.5 | 0.22 |  | 0.99 | 0.5 to 2.1 | 0.99 |  |
| Male sex | 0.95 | 0.3 to 2.7 | 0.92 |  | 0.75 | 0.4 to 1.6 | 0.44 |  |
| Orphan | 1.26 | 0.2 to 6.3 | 0.78 |  | 4.56 | 1.7 to 12.4 | 0.01 |  |
| Mother died | 3.43 | 0.6 to 20.5 | 0.18 |  | 1.27 | 0.3 to 5.9 | 0.76 |  |
| Father died | 0 | - | - |  | 0.99 | 0.2 to 4.6 | 0.99 |  |
| Edema at admission (any) | 0.71 | 0.2 to 2.6 | 0.60 |  | 0.45 | 0.2 to 1.1 | 0.08 |  |
| Edema at admission (grade 3) | 0 | - | - |  | 2.27 | 0.9 to 5.2 | 0.05 |  |
| Diarrhea at admission | 0.95 | 0.5 to 1.8 | 0.88 |  | 1.50 | 0.6 to 3.5 | 0.36 |  |
| Fever at admission | 0.80 | 0.2 to 2.9 | 0.74 |  | 1.36 | 0.6 to 3.2 | 0.48 |  |
| Cough at admission | 2.14 | 0.6 to 7.8 | 0.25 |  | 0.99 | 0.4 to 2.6 | 0.98 |  |
| MUAC at admission | 0.97 | 0.9 to 1.0 | 0.10 |  | 0.98 | 0.9 to 1.0 | 0.17 |  |
| MUAC at OTP discharge | 0.97 | 0.9 to 1.0 | 0.31 |  | 0.98 | 0.9 to 1.0 | 0.32 |  |
| MUAC <115 mm at OTP discharge | 1.87 | 0.3 to 10.6 | 0.48 |  | 2.83 | 0.8 to 9.8 | 0.10 |  |
| Attended SFP | 0.52 | 0.1 to 1.8 | 0.30 |  | 0.88 | 0.4 to 2.1 | 0.78 |  |

^a^Results of unadjusted logistic regression. CI, confidence interval; CMAM, Community-based management of acute malnutrition; MUAC,
mid-upper arm circumference; OR, odds ratio; OTP, outpatient therapeutic program; SFP, supplementary feeding program. All children were referred to SFP at discharge from OTP however not all attended (Paluku Bahwere, Angella Mtimuni, Kate Sadler, Theresa Banda, & Steve Collins, 2012)
