# Supplementary figures and images for: Relapse after severe acute malnutrition: A systematic literature review and secondary data analysis
Source: Matern Child Nutr. 2018 Oct 18;15(2):e12702. doi: 10.1111/mcn.12702 (PMC6587999; doi:10.1111/mcn.12702)

**Supplemental Figure 1.** Flow chart of literature review methods

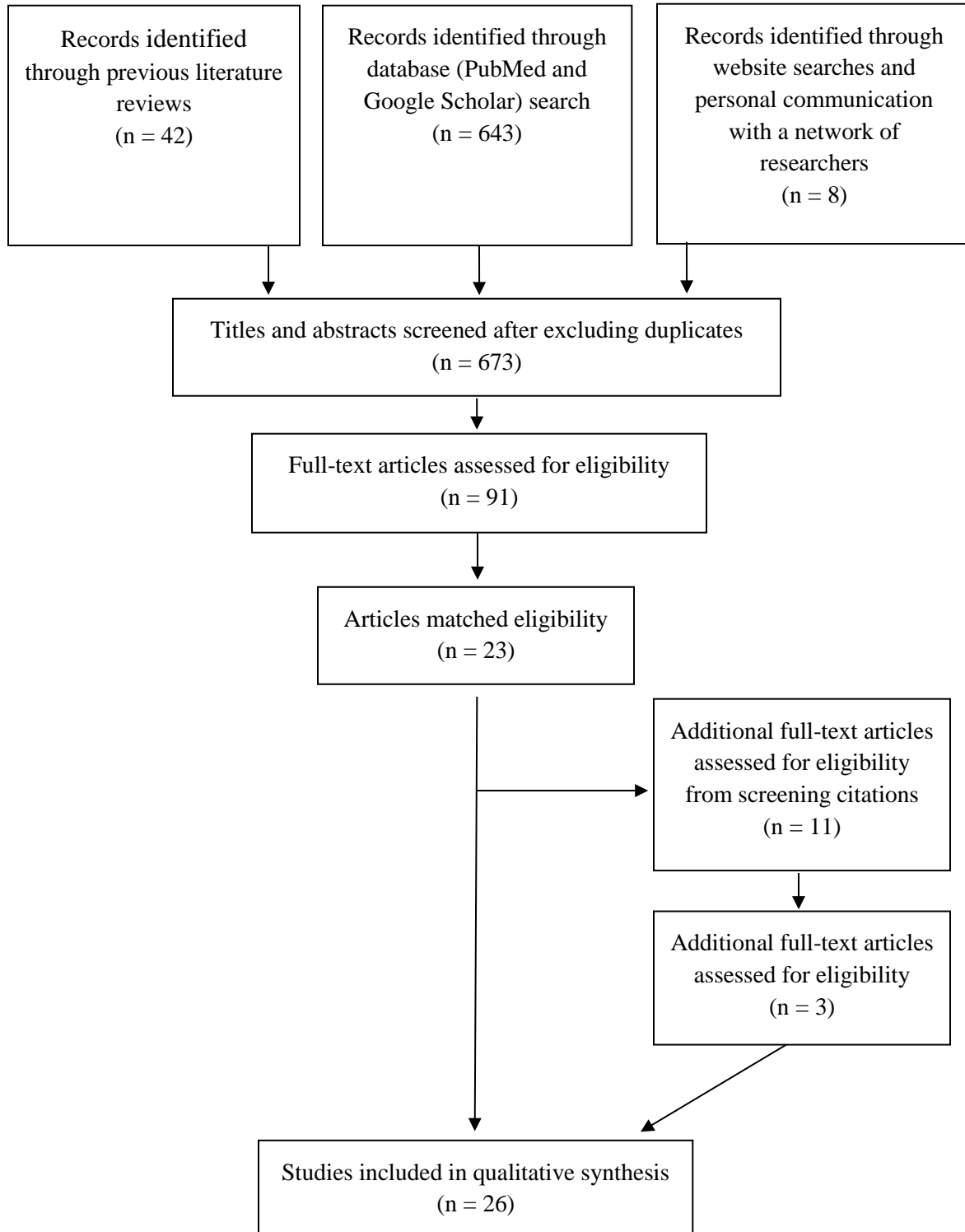

Supplement: Supplementary file 2 — Figure S1. Flow chart of literature review methods [file MCN-15-e12702-s001.pdf]
